# Targeting MET in Non-Small Cell Lung Cancer (NSCLC): A New Old Story?

**DOI:** 10.3390/ijms241210119

**Published:** 2023-06-14

**Authors:** Calogera Claudia Spagnolo, Giuliana Ciappina, Elisa Giovannetti, Andrea Squeri, Barbara Granata, Chiara Lazzari, Giulia Pretelli, Giulia Pasello, Mariacarmela Santarpia

**Affiliations:** 1 Medical Oncology Unit, Department of Human Pathology “G. Barresi”, University of Messina, 98122 Messina, Italy; spagnoloclaudia92@gmail.com (C.C.S.); giuliana.ciappina@gmail.com (G.C.); andreasqueri93@gmail.com (A.S.); barbaragranata@live.it (B.G.); 2 Department of Medical Oncology, Cancer Center Amsterdam, Amsterdam UMC, Vrje Universiteit, 1081HV Amsterdam, The Netherlands; elisa.giovannetti@gmail.com; 3 Cancer Pharmacology Lab, Fondazione Pisana per la Scienza, 56017 San Giuliano, Italy; 4 Candiolo Cancer Institute, Fondazione del Piemonte per l’Oncologia (FPO)-IRCCS, 10060 Torino, Italy; chiara.lazzari@ircc.it; 5 Department of Surgery, Oncology and Gastroenterology, University of Padova, 35128 Padova, Italy; giulia.pretelli@gmail.com (G.P.); giulia.pasello@iov.veneto.it (G.P.); 6 Oncologia Medica 2, Istituto Oncologico Veneto, IRCCS, 35128 Padova, Italy

**Keywords:** non-small cell lung cancer (NSCLC), mesenchymal-epithelial transition (MET), targeted therapy, oncogene, predictive biomarkers, tyrosine kinase inhibitors (TKIs)

## Abstract

In recent years, we have seen the development and approval for clinical use of an increasing number of therapeutic agents against actionable oncogenic drivers in metastatic non-small cell lung cancer (NSCLC). Among them, selective inhibitors, including tyrosine kinase inhibitors (TKIs) and monoclonal antibodies targeting the mesenchymal–epithelial transition (MET) receptor, have been studied in patients with advanced NSCLC with *MET* deregulation, primarily due to exon 14 skipping mutations or *MET* amplification. Some MET TKIs, including capmatinib and tepotinib, have proven to be highly effective in this molecularly defined subgroup of patients and are already approved for clinical use. Other similar agents are being tested in early-stage clinical trials with promising antitumor activity. The purpose of this review is to provide an overview of MET signaling pathways, *MET* oncogenic alterations primarily focusing on exon 14 skipping mutations, and the laboratory techniques used to detect *MET* alterations. Furthermore, we will summarize the currently available clinical data and ongoing studies on MET inhibitors, as well as the mechanisms of resistance to MET TKIs and new potential strategies, including combinatorial approaches, to improve the clinical outcomes of *MET* exon 14-altered NSCLC patients.

## 1. Introduction

Lung cancer remains the leading cause of cancer-related deaths in the world. Both molecularly targeted therapy and immunotherapy have revolutionized the therapeutic landscape of this heterogenous disease, which includes different histologic and molecular subtypes. Non-small cell lung cancer (NSCLC) accounts for the majority of lung cancer cases. In the last decade, we have assisted in the development and approval for clinical use of an increasing number of therapeutic agents against actionable oncogenic drivers in advanced or metastatic NSCLC. In this setting, currently, the National Comprehensive Cancer Network (NCCN) guidelines recommend testing specific biomarkers to select the most appropriate first-line treatment strategy that, in those patients harboring oncogenic alterations, including mutations of the epidermal growth factor receptor (*EGFR*) and v-raf murine sarcoma viral oncogene homolog B1 (*BRAF*) or rearrangements of anaplastic lymphoma kinase (*ALK*) or c-ros oncogene 1 (*ROS1*), consists of targeted therapies (www.nccn.org NCCN guidelines NSCLC version 3 2022 accessed on 14 May 2023) [1].

For patients without driver alterations and high programmed cell death-ligand 1 (PD-L1) expression [tumor proportion score (TPS) ≥ 50%], immunotherapy has become the standard of care, as reported by updated ESMO guidelines, while a combination of chemotherapy and immunotherapy is recommended for most patients with negative or low PD-L1 expression [2].

Recently, emerging biomarkers, including genetic alterations of the Kirsten rat sarcoma viral oncogene homolog (*KRAS*), the human epidermal growth factor receptor 2 (*HER2*), the mesenchymal-epithelial transition (*MET*) and rearrangements of the neurotrophic tyrosine receptor kinase (*NTRK*) and the rearranged during transfection (*RET*) genes, have gained significant attention because of the development of selective inhibitors, some of which have already received approval for clinical use in pretreated NSCLC patients [3,4].

MET-directed therapies, including tyrosine kinase inhibitors (TKIs) and monoclonal antibodies targeting MET, MET ligands, or the hepatocyte growth factor (HGF) [5,6,7], have been tested in patients with advanced NSCLC with MET deregulation, mainly due to exon 14 skipping mutations (*MET*ex14) or MET amplification (*MET*-amp).

Currently, two selective MET TKIs, capmatinib and tepotinib, have been demonstrated to be highly effective in this molecularly defined subgroup of patients, as showed in the GEOMETRY Mono-1 and VISION trials, respectively, and have obtained FDA and EMA approval. Moreover, other similar agents are being tested in early-phase clinical trials with promising anti-tumor activity [7,8,9] (www.FDA.gov accessed on 14 May 2023).

This review will provide an overview on the biology of MET signaling pathways, on *MET* alterations, mainly focusing on exon 14 skipping mutations, and techniques used to detect *MET* alterations. We will summarize currently available clinical data and ongoing trials on MET inhibitors, as well as mechanisms of resistance and novel strategies to improve long-term clinical outcomes.

## 2. HGF/MET Signaling Pathway

The c-*MET* proto-oncogene is located on chromosome 7q21-q31 [10]. Its transcription is regulated by several factors, such as E-Twenty Six (ETS), paired box 3 (Pax3), activator protein 2 (AP2) and transcription factor 4 (Tcf-4). The transcription product of this gene is the protein tyrosine kinase MET, a transmembrane receptor belonging to the family of receptor tyrosine kinases (RTKs) that recognizes the hepatocyte growth factor (HGF) as a ligand and regulates several essential cellular processes. This receptor is expressed on the epithelial cells of many organs, including the liver, pancreas, prostate, kidney, muscle and bone marrow, both during embryogenesis and in adulthood [11].

Similarly to other RTKs, MET encompasses different domains: an extracellular domain, a transmembrane domain, and an intracellular portion consisting of a juxtamembrane domain, a catalytic kinase domain and a carboxy-terminal tail C (C-tail). The extracellular portion of MET is made up of three types of domains: the N-terminal segment, folded to form a large semaphorin-like (SEMA) domain including the whole alpha subunit and part of the beta subunit; the plexin-semaphorin-integrin (PSI) domain which follows the SEMA and includes four disulfide bridges; in turn, the PSI is connected with four immunoglobulin-plexin-transcription factor (IPT) domains [12].

The stability and degradation of the MET receptor are regulated by the intracellular juxtamembrane domain, encoded by exon 14 of the gene, containing a tyrosine residue (Y1003). The phosphorylated Tyr1003 works as a binding site for the casitas-B-lineage lymphoma (CBL) E3 ubiquitin ligase that is able to ubiquitylate MET leading to internalization of the receptor from the cell membrane to endocytic vesicles and subsequently to lysosome-mediated degradation [13,14].

The c-MET ligand was primarily identified both as a factor regulating motility and as a growth factor for hepatocytes, and only later recognized as the same molecule: the hepatocyte growth factor (HGF), also known as scatter factor (SF) [15]. HGF acts as a pleiotropic factor and a cytokine, promoting cell proliferation, survival, motility, differentiation and morphogenesis [16]. This factor is secreted by mesenchymal cells in the form of a biologically inert, single-chain precursor and is converted into its bioactive form by the action of extracellular proteases that cut the bridge between Arg494 and Val495. The mature form of HGF consists of an alpha chain and a beta chain, held together by a disulfide bridge [11]. The binding of HGF to c-MET leads to homodimerization and phosphorylation of the receptor, at the level of two tyrosine residues (Y1234 and Y1235) located on the catalytic loop of the tyrosine kinase domain [17]. This results in the tyrosines 1349 and 1356 being phosphorylated in their carboxy-terminal part. When this occurs, the phosphorylated tyrosines recruit signaling effectors including growth factor receptor-bound protein 2 (GRB2), src homology-2-containing protein (SHC), phosphatidylinositol 3-Kinase (PI3K), and Signal Transducer And Activator Of Transcription 3 (STAT-3) [18]. The activation of c-MET signaling is common to many RTKs. Indeed, MET activates RAS, which leads to activation of the RAF kinase that subsequently activates effector MEK and the mitogen activated protein kinase (MAPK) cascade [19].

Other downstream pathways of c-MET are the PI3K/AKT, the STAT and the NF-κB pathways, involved in cell proliferation, differentiation, cell motility, invasion and survival. The p85 subunit of PI3K can bind c-MET directly or indirectly via GAB1, which in turn results in AKT/protein kinase B activation. This axis is responsible for c-MET signaling-mediated cell survival [20]. Moreover, by regulating cell-matrix adhesion and promoting cytoskeletal changes and cell migration, MET plays a key role in epithelial to mesenchymal transition (EMT) [21,22].

Alterations of the MET pathway are responsible for the tumorigenesis of many solid neoplasms. In NSCLC, this dysregulation occurs through mechanisms of gene mutation, gene amplification, rearrangement and protein overexpression [23].

## 3. MET Alterations

### 3.1. MET Exon 14 Skipping Mutation

Several alterations of *MET* exon 14, such as point mutations, insertions and deletions have been identified in patients with NSCLC [24]. As commented above, exon 14 of the gene encodes part of the juxtamembrane domain of the receptor, containing a tyrosine residue (Y1003) bound by the CBL E3 ubiquitin ligase that determines the internalization and degradation of the receptor [13].

Point mutations are the most common alterations (49.5%) and are responsible for exon 14 skipping during pre-mRNA splicing, leading to the loss of the CBL-binding site and increasing the half-life of the MET receptor [25]. Furthermore, the MET Y1003 mutation leads to a decrease in ubiquitination and increased stability of the MET protein [26]. Historically, the technique used for the identification of *MET* exon 14 mutations was immunohistochemistry (IHC); however this has proved to be ineffective in clinical practice as it is characterized by a non-negligible rate of false negative results. FISH (fluorescence in situ hybridization) is used to identify copy number variations but, in the case of MET alterations, this method is burdened by a large laboratory-dependent variability. Real-time polymerase chain reaction (RT-PCR) is a low-cost method characterized by a high diagnostic sensitivity and therefore widely used in clinical practice to identify this mutation. However, the fragmentation of nucleic acids can invalidate the use of this technique in cases of scarce tissue samples. To date, the most useful method to detect *MET* exon 14 skipping mutations is next generation sequencing (NGS), which is characterized by a higher sensitivity. As for the detection of other gene alterations in NSCLC and also for *MET* exon 14 skipping, different NGS assays can be used, based on the analysis of DNA or RNA extracted from tumor tissue. Targeted amplicon-mediated DNA-based NGS is widely used in clinical practice; however, it may not be able to identify a part of the *MET* alterations, due to the high variety and specific localization of these mutations [27]. Another possibility is the use of RNA-based NGS methods. These have demonstrated a higher detection rate for *MET* exon 14 skipping than the amplicon/DNA-based, being able to identify this alteration even in a significant proportion of DNA-sequencing driver-negative cases [28]. Despite the greater sensitivity of RNA-based methods, there are some limitations associated to their use in clinical routine practice, such as the inadequacy of the tumor tissue material for RNA extraction, with consequent failure of the method in a non-negligible percentage of cases (about 30%) [29]. MET alterations can be also detected in circulating tumor DNA (ctDNA) from plasma samples with less sensitivity compared to tissue analysis, probably depending on tumor shedding [30]. However, in the case of tumor tissue not being available for biomarker identification or if the analyses carried out on the tissue should prove inconclusive, liquid biopsy can represent a valid alternative. As demonstrated by the VISION study, the use of plasma hybrid capture-based NGS with common commercial assays can accurately detect METex14 on ctDNA, with satisfactory concordance rates compared to analyses performed on tumor samples [9].

Finally, a recent retrospective analysis conducted on 4233 patients affected by NSCLC with *MET* exon 14 skipping undergoing NGS both on tumor tissue and on plasma samples, allowed the identification and classification of new variants of this alteration, using the in silico SpliceAI tool for skipping prediction. This method can help to define as skipping variants those with a delta score above 0.315 with a determination ratio of 0.92% [31]. New and standardized tools are needed for the correct identification of *MET* exon 14 skipping mutations and its variants, to allow for the best therapeutic choice.

Regarding clinical and pathological features of MET-altered tumors, the *MET* exon 14 skipping mutation is more frequent in the adenocarcinoma histotype and in elderly patients (median age 77 years) with a history of smoking [32]. However, this alteration can also be found in squamous NSCLCs, with a prevalence of up to 9% in most recent trials [8]. De novo alterations are often mutually exclusive with other driver mutations such as *KRAS*, *EGFR*, *ALK*, *ROS1*, and *RET* and frequently coexist with the *TP53* mutation [33]. The presence of the *MET* exon 14 skipping mutation has been associated with marked sensitivity to MET tyrosine kinase inhibitors (TKIs) [8,9].

### 3.2. MET Amplification

Another mechanism of oncogenic activation of *MET* is amplification of the wild-type gene located on chromosome 7 (MET-amp). *MET*-amp can be evaluated by two different methods. The first is based on the identification of the gene copy number (GCN). *MET*-amp is defined as the presence of five or more copies of *MET* per cell (MET GCN ≥ 5). However, this method does not distinguish the amplification from polysomy [34]. With the use of Fluorescent in situ hybridization (FISH), the amplification of MET can be measured by estimating the ratio between the number of copies of MET and the copy number of the centromere of chromosome 7 (CEP7). This method distinguishes *MET* amplification (gene copy number increase) from *MET* polysomy (chromosome 7 copy number increase) [35].

In a study by Noonan et al. there is a distinction between low (1.8–2.2), intermediate (2.2–5) and high (>5) ratios [36]. Commonly, in *MET*-amp tumors, the MET/CEP7 ratio is high. The presence of one of these criteria identifies *MET*-amp tumors: MET/CEP7 ratio ≥1.8; >10% of cells with clusters of MET signals; or *MET* copy ≥15. Polysomy 7 was considered with MET/CEP7 ratio <1.8 and MET copy ≥5 but <15 [37].

With NGS, identification of *MET* amplification depends on the platform used. In general, the sequenced gene of interest is normalized against a comparator such as archived sequences from normal samples [34]. However, while NGS is being increasingly used in clinical practice, blood-based NGS testing has lower sensitivity than tissue analysis and may underestimate gene amplification [38]. Standardized methods are necessary in clinical practice to identify gene amplification. However, in lung tumors with high MET/CEP7 ratio, this seems to represent a predictor of response to MET TKIs and appears to identify a subset of tumors with a greater dependence on MET signaling [39]. *MET* amplification is recognized as a de novo driver alteration in approximately 1–5% of NSCLC, and is usually typical of heavy-smoking patients [40]. Moreover, MET-amp seems to be associated with a high percentage of co-occurring mutations (mostly *TP53*, *KRAS*, and *KEAP1*) [41].

Finally, *MET*-amp is often associated with acquired resistance to common EGFR TKIs, in NSCLC patients with *EGFR*-sensitizing mutations [42].

### 3.3. MET Overexpression

MET overexpression has a variable incidence (about 35% to 72%) in NSCLC, depending on the antibody used for detecting it [43]. With immunohistochemistry (IHC) methods, MET expression can be identified by a score between 0 and 3+, based on antibody SP44 binding [44]. The most common cause of MET overexpression is transcriptional upregulation of the MET protein receptor in the absence of gene amplification [45]. High MET expression levels have been associated with a poor prognosis, but its role as a predictive factor is not clear in the absence of other MET alterations [23]. In fact, several phase III trials evaluating MET inhibitors in NSCLC with MET alterations including MET overexpression showed a limited efficacy in this subset of patients [46,47].

### 3.4. MET Fusion

One way for the oncogenic activation of the MET pathway is the formation of fusion genes through chromosomal translocation mechanisms. In NSCLC, the tyrosine kinase domain-encoding portion of *MET* creates a fusion gene with a wide variety of partners [48], including *TPR*, *KIF5B*, *HLA-DRB1* [49,50,51], making the receptor constitutively activated. MET fusions account for only 0.2% to 0.3% of lung cancer patients and about 50% are intragenic fusions. The techniques used for the identification of gene fusions include IHC, FISH, RT-PCR and NGS. FISH is a low-cost and widely available method and it identifies most *MET* fusions, but does not allow the identification of intragenic fusions. As far as IHC is concerned, the major limit is given by false positivity. Indeed, many cases, identified as IHC positivity, have been demonstrated to be FISH- and NGS negative, suggesting the hypothesis that IHC led to the misleading detection of mutations other than fusions (e.g., amplifications) [52]. Although RT-PCR is an effective, manageable and available method, to date RNA NGS or combined DNA/RNA NGS is the method of choice for the identification of *MET* gene fusions [53]. Despite the potential predictive role of these alterations, the first data demonstrated that only few isolated cases of NSCLC with *MET* fusions achieved benefits from treatment with MET inhibitors [48].

**Figure 1:**
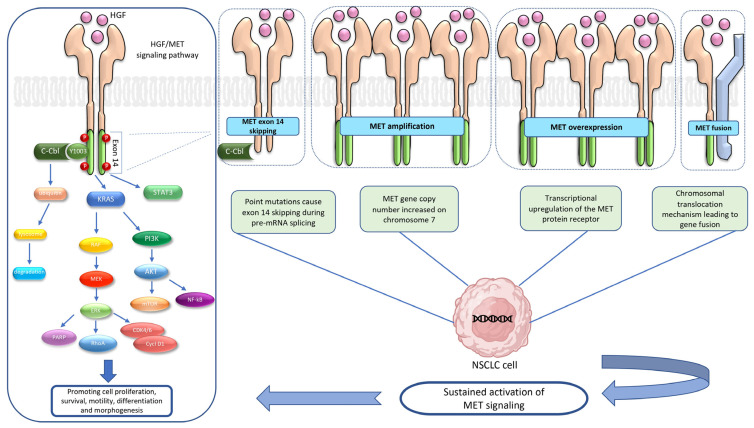
HGF/*MET* signaling pathway and most frequent alterations in NSCLC. The binding of HGF to MET leads to homodimerization and phosphorylation of the receptor that, with a cascade mechanism, activates downstream pathways such as RAS, PI3K/AKT, the STAT and NF-κB, involved in cell proliferation, differentiation, cell motility, invasion and survival. Furthermore, the stability and degradation of the MET receptor are regulated by the intracellular juxtamembrane domain, encoded by exon 14 of the gene, containing a tyrosine residue (Y1003). The phosphorylated Tyr1003 works as a binding site for the casitas-B-lineage lymphoma (CBL) E3 ubiquitin ligase that is able to ubiquitylate MET leading to internalization and degradation of the receptor. In NSCLC, point mutations of the *MET* gene can cause skipping of exon 14 during pre-mRNA splicing, leading to the loss of the CBL-binding site and increasing the half-life of the MET receptor. Increasing of *MET* gene copy numbers on chromosome 7 causes *MET* amplification while an upregulation of the MET protein receptor in the transcription phase leads to MET overexpression. Both these mechanisms can lead to a global increase in MET signaling. Finally, chromosomal translocation is responsible for the creation of a fusion gene with a wide variety of partners, such as *TPR*, *KIF5B* and *HLA-DRB1*, making the receptor constitutively activated. Abbreviations: AKT, protein kinase B; C- CBL, casitas-B-lineage lymphoma E3 ubiquitin; CDK, cyclin-dependent kinase; Cycl D1, cyclin D1; ERK, extracellular signal-regulated kinase; HGF, hepatocyte growth factor; KRAS, Kirsten rat sarcoma viral oncogene; MEK, mitogen-activated protein kinase; MET, mesenchymal epithelial transition; mTOR, mammalian target of rapamycin; NSCLC, non-small-cell lung cancer; NF-κB, nuclear factor κB; PARP, poly (ADP-ribose) polymerases; PI3K, phosphatidylinositol 3-kinase; RAF, rapidly accelerated fibrosarcoma; RhoA, ras homolog gene family, member A; STAT 3, signal transducer and activator of transcription 3.

Moreover, although data are still limited given the rarity of this alteration, *MET* gene fusions could represent a mechanism of resistance to EGFR or ALK-TKIs [31] (Figure 1).

## 4. Targeting the HGF/MET Axis

Preclinical studies showed that MET blockade was effective only in those tumors harboring *MET* genomic alterations, while targeting the HGF/MET pathway in tumors with wild type MET had a poor effect on cancer cell proliferation [6,54,55]. Several therapeutic strategies have been developed to target MET signaling, including anti-MET- or anti-HGF-antibodies and small molecule TKIs.

### 4.1. MET TKIs

MET TKIs are small molecules that can generally be subdivided into three groups, based on their structure and on their binding with MET. Type I and II are both ATP-competitive inhibitors and are those mainly being analyzed in clinical trials. Type I inhibitors fill the ATP-binding pocket and bind to the activation loop (A-loop), with Y1230, blocking catalytic activation. This type is also subdivided into type Ia and type Ib. Type Ia inhibitors, such as crizotinib, interact with MET by the solvent front residue G1163 and are also known as non-selective MET TKIs because this residue is not specific to MET. Type Ib inhibitors, such as capmatinib, tepotinib, savolitinib, and APL-101, selectively interact with MET alone, by the Y1230 residue. Type II MET TKIs, such as cabozantinib, foretinib, merestinib and glesatinib, are also ATP-competitive inhibitors. They fill the ATP-binding pocket and bind to the inactive “DFG out” conformation of MET. Therefore, their binding is independent of interactions with the A-loop. Type III MET TKIs are non-ATP competitive inhibitors, binding allosterically outside the ATP pocket [6,7,23]. An overview of the main published clinical trials with MET TKIs in NSCLC is shown in Table 1**.**

#### 4.1.1. Multitargeted or Non-Selective MET TKIs

##### Crizotinib

Crizotinib is an oral, small-molecule inhibitor of several kinases such as MET, ALK, ROS1 and RON. Currently, it is approved for the treatment of advanced NSCLC characterized by *ALK* or *ROS1* rearrangements [56,57]. This drug can act by binding and blocking the ATP-binding site of RTK, thus inhibiting the activation of crucial intracellular signaling pathways. Use of crizotinib in tumor cells characterized by *MET* exon 14 skipping mutations decreases the viability of tumor cells and blocks related downstream signaling [58]. Several clinical trials showing that the use of crizotinib is associated with clinically significant responses in patients with advanced NSCLC with *MET* exon 14 alteration have been performed [58,59,60,61,62,63,64]. The phase I trial, PROFILE 1001, evaluated the role of treatment with crizotinib in patients with advanced NSCLC characterized by several genetic alterations including those related to *MET*. A total of 69 patients with *MET* exon 14 skipping mutations were enrolled in the study and 65 of these were evaluable for tumor response. The objective response rate (ORR) was 32% (95% CI 21–45), median progression free survival (PFS) 7.3 months (95% CI 5.4–9.1) and median duration of response (DOR) 9.1 months (95% CI 6.4–12.7) [65]. In addition, 38 of the patients enrolled in this study, characterized by high level *MET* gene amplification by FISH (MET to CEP7 ratio ≥4) showed increased median PFS with crizotinib compared to patients characterized by intermediate- (MET/CEP7 ratio >2.2 to <4) or low- (MET/CEP7 ratio >1.8 to <2.2) level *MET* amplification (median PFS 6.7 months vs. 1.9 months vs. 1.8 months, respectively) [66]. The most common treatment-related adverse events (TRAEs) were represented by peripheral edema (found in 51% of patients), gastrointestinal symptoms and fatigue [65,67]. TRAEs of grade ≥3 were found in 29% of patients and were mainly represented by increased serum transaminases levels (4%) and dyspnea (4%), while development of a fatal interstitial pneumonia was found in only 1 patient [65]. Based on the results of this study, in 2018 the FDA granted breakthrough therapy designation for crizotinib for the treatment of patients with NSCLC with *MET* exon 14 alterations with disease progression on or after platinum-based chemotherapy (www.fda.gov accessed on 14 May 2023). A phase II clinical trial, the METROS study, analyzing the efficacy of crizotinib in patients with *MET* exon 14 skipping mutations and amplification, showed limited clinical benefit, with an ORR of 27% (95% CI 11–47) and a median PFS of 4.4 months (95% CI 3.0–5.8) [68]. Another phase II clinical trial, the AcSé trial, has shown similar results in NSCLC patients with *MET* alterations. In the c-*MET* ≥6 copies cohort, ORR was 16% and PFS 3.2 months while in all c-*MET*-mutations cohorts ORR was 10.7% and PFS 2.2 months [69]. In addition, there are other phase II ongoing clinical trials evaluating the effectiveness of crizotinib in advanced refractory NSCLC characterized by *MET* amplification and *MET* exon 14 skipping mutation and also in patients with NSCLC with *ROS1* fusion (NCT02664935, NCT02465060, NCT04084717).

##### Cabozantinib

Cabozantinib (XL-184) is another small-molecule, non-selective type II MET TKI, able to target several kinases, including VEGFR1–3, RET, TIE2, FLT-3 and KIT. Currently, cabozantinib is approved for treatment of several cancers such as medullary thyroid carcinoma, renal cell carcinoma and hepatocellular carcinoma (www.fda.gov, accessed on 14 May 2023). The COSMIC-313 (NCT03937219) phase III clinical trial recently reported that among patients with previously untreated advanced renal-cell carcinoma and intermediate or poor prognostic risk, the combination of cabozantinib with nivolumab and ipilimumab led to significantly longer PFS compared to treatment with nivolumab and ipilimumab alone. However, it is important to note that the experimental group experienced a higher incidence of grade 3 or 4 adverse events compared to the control group [70].

Pre-clinical in vivo data have shown that cabozantinib can also be effective against other cancers such as lung cancer [71]. Moreover, a phase II trial has shown that *EGFR* wild-type patients with advanced NSCLC treated with cabozantinib alone or in combination with erlotinib had improved PFS (cabozantinib alone 4.3 months, HR 0.39, 80% CI 0.27–0.55; cabozantinib and erlotinib 4.7 months, HR 0.37, 80% CI 0.25–0.53) compared to erlotinib alone (1.8 months, 95% CI 1.7–2.2) [72]. However, although the trial has shown promising results, this has not evaluated the effectiveness of this drug in the presence of *MET* alterations and it is still not now fully clear if cabozantinib treatment can play a role in patients with *MET* alterations. In an initial study enrolling patients with stage IV NSCLC with *MET* exon 14 skipping mutations and treated with crizotinib or cabozantinib, Paik et al reported a complete response in one patient treated with cabozantinib [58].

Several phase II clinical trials are ongoing to evaluate the effectiveness and safety of cabozantinib in the treatment of metastatic NSCLC with *MET* alteration (NCT01639508, NCT05613413, NCT04310007, NCT03911193).

##### Merestinib

Merestinib (LY2801653) is an oral ATP-competitive, multitargeted inhibitor of MET and other kinases involved in tumor cell growth, proliferation, and angiogenesis, such as AXL, ROS1, VEGFR2, FLT3, DDR1–2, MERTK and NTRK1–3. It was characterized by a manageable safety profile and potential antitumoral activity in pre-treated advanced cancer patients [73,74]. In preclinical in vitro and in vivo NSCLC models, this drug showed a significant inhibition of tumor growth in tumor cell lines and patient-derived tumor xenograft models as a single agent or when used in combination with other antineoplastic agents. Furthermore, in a metastatic lung orthotopic tumor model, Merestinib treatment conspicuously reduced both primary tumor growth and metastasis [75]. Indeed, this TKI decreases the phosphorylation of important downstream proteins, such as CBL, PI3K and STAT3, leading to the inhibition of tumor growth in mouse xenograft models of lung cancer [76]. Merestinib showed to be effective when used as single agent or when in combination with the bivalent anti-MET antibody, emibetuzumab, in a gastric cell line and xenograft model harboring *MET* exon 14 mutations (*MET* exon14 skipping and *MET* amplification) [73]. An ongoing phase II clinical trial is evaluating the activity of merestinib in NSCLC harboring *MET* exon 14 skipping mutations or advanced solid cancers with *NTRK1–3* rearrangements (NCT02920996).

##### Glesatinib

Glesatinib is an oral ATP-competitive, multitargeted TKI of MET, VEGFR1–3, AXL, RON and TIE2. It is a unique type II MET inhibitor, and interacts with MET independently of the binding of A-loop, demonstrating its activity against *MET* mutations involving the residues D1228 and Y1230, which confer resistance to type I MET TKIs. Glesatinib was shown to be effective in tumor cell lines and exerted a significant regression of cell- and patient-derived tumor models harboring *MET*ex14 del mutation in vivo [77].

However, significant clinical data with this TKI are still missing. A phase I trial of glesatinib in advanced, pre-treated, NSCLC patients with activating genetic *MET* alterations (mutation or amplification) has shown that the glesatinib safety profile was acceptable and the dosage of 750 mg twice daily (NCT00697632) [78] was selected for the subsequent phase II clinical trial (NCT02544633) evaluating glesatinib efficacy. In addition, another phase II ongoing clinical trial is evaluating glesatinib treatment in combination with the anti-PD-1 nivolumab in advanced NSCLC (NCT02954991).

#### 4.1.2. Selective MET TKIs

##### Capmatinib

Capmatinib (INC280) is an oral, ATP-competitive, type Ib MET TKI, which demonstrated high selectivity in MET-driven mouse tumor models [79].

Significant antitumor activity was seen in cell-line- or patient-derived xenograft models with different mechanisms of MET activation, including *MET* exon 14 alterations. Notably, enhanced antitumor activity of capmatinib was found in those models in which MET activation occurred concomitantly with other oncogenic alterations, such as *EGFR* mutations, when used in combination with EGFR TKIs [80].

In May 2020 Capmatinib gained FDA accelerated approval for patients with metastatic NSCLC with *MET* exon 14 skipping mutation (www.fda.gov, accessed on 14 May 2023). This was based on the results of the GEOMETRY mono-1 (NCT02414139), a multicenter, non-randomized, open-label, multi-cohort, phase 2 trial evaluating capmatinib (400 mg orally twice daily) in *EGFR* wild-type and *ALK*-negative NSCLC patients with *MET* alterations, including *MET* exon 14 skipping mutation or *MET* amplification. Patients were stratified based on MET status and prior lines of therapy. Increased ORR and PFS were observed in treatment-naïve patients compared to pretreated patients. Indeed, in the group of previously treated patients with *MET* exon 14 skipping mutations, the ORR and DoR were 41% and 9.7 months, respectively, while in the naïve patients’ group these were 68% and 12.6 months, respectively. In addition, an increased median PFS was found in naïve patients (12.4 months) compared to previously treated patients (5.4 months). This study also demonstrated the efficacy of capmatinib in patients with *MET* amplification and GCN ≥10 NSCLC, although to a lesser extent than in patients with *MET* exon 14 alterations.

In addition, this drug exhibits intracranial activity and an acceptable safety profile, as most of the TRAEs were of grade 1/2 and were represented by peripheral edema, nausea, vomiting, and increased serum creatinine levels. The treatment was discontinued in 11% of patients and a dose reduction occurred in 23% of patients.

Several phase 2 clinical trials of capmatinib in patients with *MET* exon 14 mutation-positive NSCLC are ongoing (NCT03693339, NCT04677595, NCT05567055). The phase 2 Geometry-N trial (NCT04926831) is evaluating the activity of capmatinib in adjuvant and neoadjuvant settings. Furthermore, a phase 3 study (NCT04427072) investigating capmatinib versus docetaxel in previously treated patients with *MET* exon 14 skipping NSCLC is still ongoing, while two clinical trials evaluating the safety and efficacy of capmatinib in combination with immunotherapeutic agents (spartalizumab and pembrolizumab) were recently terminated due to lack of tolerability observed in the combination treatment group when compared to data from single agent studies (NCT04323436, NCT04139317).

##### Tepotinib

Tepotinib is an oral, type Ib MET inhibitor, acting as an ATP-competitive, and highly selective for MET. In vivo, tepotinib has been shown to be able to induce tumor regression in a human-cancer mice model, irrespective of whether or not MET activation was dependent on HGF [6,81]. In addition, tepotinib has shown promising results when used in patients with MET-driven tumors [82]. The FDA has approved the use of tepotinib for the second-line treatment for patients affected by NSCLC carrying *MET* exon 14 skipping mutations based on the results of the phase II VISION trial. In this trial, the use of tepotinib in advanced NSCLC patients with *MET* alterations, including *MET* exon 14 skipping mutations and *MET* amplification, was correlated with an ORR of 46% (95% CI 36–57) based on an independent review, and a median PFS of 8.5 months (95% CI 6.7–11). Also, there were no significantly differences in terms of response between previously treated and naïve patients [9]. TRAEs of grade 3 or higher were reported in 28% of the patients and peripheral edema was the most common side effect observed in 7% of patients which led to a dose reduction in 16% of the patients and a dose interruption in 18%, while permanent discontinuation was uncommon (5%) [9,67]. Moreover, in the VISION trial the efficacy and safety of tepotinib in a sub-group of elderly patients (over 75 years old) with NSCLC characterized by *MET* exon 14-altered resulted similar to the other enrolled patients (ORR 39.7%, 95% CI 28–52.3; median PFS 8.6 months, 95% CI 6.9–12.4) [9]. In this sub-group, the most common side effect was peripheral edema found in 51.4% of the elderly patients. Grade 3 or higher TRAEs were found in 34% of patients and 14.7% of patients have discontinued treatment [9]. Several phase 1 and 2 clinical trials are ongoing (NCT04739358, NCT03940703, NCT05782361) in order to assess the safety and effectiveness of tepotinib in advanced NSCLC with *MET* alterations.

##### Savolitinib

Savolitinib is an oral, ATP-competitive, type Ib MET TKI, with high selectivity for MET, approved on June 2021 in China for patients with advanced NSCLC with *MET* exon 14 skipping alterations with progression disease after prior systemic therapy or who are unable to receive chemotherapy [83,84]. This was based on data from a multicenter, multicohort phase 2 study evaluating the efficacy and safety of savolinitib in unresectable or metastatic NSCLC with *MET* exon 14 altered NSCLC or metastatic pulmonary sarcomatoid carcinoma (PSC) in Chinese patients [85]. Savolitinib was administered at either 600 mg once daily (in patients with body weight ≥50 kg) or 400 mg once daily (body weight <50 kg). The primary endpoint was ORR. Results showed an ORR of 49.2% (95% CI 31.1–55.3) in the tumor response evaluable set with similar response rates independently of histologic subtype (ORR 40.0% in PSC versus 44.4% in other NSCLC subtypes) or previous treatment (ORR 46.4% in first-line setting versus 40.5% in subsequent line settings). The median PFS was 6.9 months while the median overall survival (OS), at the final analysis, was 12.5 months [85,86]. To note, patients with PSC showed worse outcomes compared to non-PSC patients with a median OS of 10.6 months (95% CI, 4.6–14.9) and 17.3 months (95% CI, 10.6–23.6), respectively [86]. The most common TRAEs were peripheral edema (56%), nausea (53%), hypoalbuminemia (41%), increased serum liver enzymes levels (ALT and increased aspartate aminotransferase AST, 39%). Grade 3 or higher TRAEs were reported in 46% of patients [85]. Furthermore, clinical trials evaluating the efficacy of savolitinib in locally advanced or metastatic NSCLC patients with *MET* alterations are still ongoing (NCT04923945), also in combination with chemotherapeutic drugs (docetaxel) (NCT05777278) or immunotherapeutic agents (durvalumab) (NCT05374603). Additionally, savolitinib is being studied in patients with MET-driven resistance to EGFR TKIs in combination with osimertinib (SAVANNAH, NCT03778229; SAFFRON, NCT05261399; ORCHARD, NCT03944772; NCT03944771; SACHI, NCT05015608) and further in *EGFR*-mutant NSCLC patients with de novo *MET* positive alterations as first-line setting (FLOWERS, NCT05163249; SANOVO, NCT05009836) (www.clinicaltrial.gov, accessed on 14 May 2023).

Other MET TKIs are being tested in ongoing clinical trials such as APL-101 (SPARTA, NCT03175224), elzovantinib or TPX-0022 (SHIELD-1, NCT03993873), and Glumetinib or SCC244 (GLORY, NCT04270591). To note, the first report of the data of the ongoing single-arm phase Ⅱ study trial GLORY (NCT04270591) showed promising results for Glumetinib (SCC244), with an ORR by BIRC of 60.9% overall, and 66.7% and 51.9% in treatment-naïve and previously treated patients, respectively. The median duration of response (DoR) was 8.2 months and the median PFS was 7.6 months [87]. An overview of the ongoing clinical trials with MET TKIs in NSCLC is shown in Table 2.

### 4.2. MET Antibodies 

In addition to MET TKIs, there are different antibodies targeting MET that have been studied or that are in development for the treatment of NSCLC patients with *MET* alterations. These antibodies inhibit the MET signaling pathway by blocking interactions between the MET receptor and its ligand HGF.

#### 4.2.1. Anti-MET/HGF Antibodies

These antibodies can be categorized into anti-MET antibodies, including onartuzumab and emibetuzumab, and anti-HGF antibodies, such as rilotumumab and ficlatuzumab. The efficacy of specific anti-MET monoclonal antibodies has been shown to be unsatisfactory in clinical trials. A phase 3 study of erlotinib with or without onartuzumab in previously treated patients with advanced *MET*-positive NSCLC (defined as ≥50% of tumor cells with MET IHC scores of 2+ or 3+) resulted in early study termination, because it showed worse survival rates in the experimental group than in the placebo group, failing to demonstrate significant clinical efficacy [47]. A phase 2 study of erlotinib with or without emibetuzumab in patients with advanced *EGFR*-mutated NSCLC demonstrated no difference in clinical outcomes in the intention-to-treat population [88]. However, exploratory post hoc analysis showed improved PFS in the combination group compared to erlotinib monotherapy (median PFS 20.7 vs. 5.4 months; HR 0.38; 90% CI, 0.17–0.91) among patients with MET IHC of 3+ in ≥90% of tumor cells. These results provided data on the potential benefit of anti-MET antibodies when limited to patients with high-level MET expression [88]. On the other hand, novel agents were studied. These are bispecific antibodies with dual antigenic targeting mechanisms, with a higher tumor selectivity and decreased toxicity [89].

Amivantamab is a monoclonal antibody with dual activity (anti-EGFR and anti-MET) but with a higher affinity for MET (40 pM) than EGFR (1.4 nM). In NSCLC with *EGFR* exon 20 insertion mutations showing intrinsic resistance to approved TKIs, for which the standard of care remains platinum-based chemotherapy with an associated reduced median overall OS of 16 months [90], amivantamab, by its capacity to bind to the extracellular part of each receptor domain, is able to bypass the resistance at the binding site of the TKI, demonstrating clinically meaningful efficacy in these patients [91]. Therefore, it received accelerated FDA approval in May 2021 for patients with NSCLC with *EGFR* exon 20 insertions who progressed on or after platinum-based chemotherapy (www.fda.gov accessed on 14 May 2023).

Results from the ongoing phase 1 CHRYSALIS study, evaluating amivantamab in combination with lazertinib (a third-generation EGFR TKI) for the treatment of osimertinib-relapsed, chemotherapy-naïve *EGFR*-mutant NSCLC, showed promising effectiveness and an acceptable safety profile (CHRYSALIS, NCT02609776). In a dose-expansion cohort including 43 patients with *MET* exon 14 skipping mutations, an ORR of 33%was reported. The response rate was impacted by prior therapy (treatment-naïve: ORR 50%; no prior MET inhibitor: ORR 45.5%; prior MET inhibitor, ORR 21.1%). The median PFS was 6.7 months [92]. A low incidence of grade ≥3 TRAEs occurred, including rash (4%), infusion-related reaction (3%) paronychia (1%) and hypoalbuminemia (3%). Results also showed improved central nervous system (CNS) protection with the combination approach (progression rate of 17% with single agent amivantamab compared to 7% with amivantamab plus lazertinib) [92].

In addition, other trials evaluating the combination of amivantamab plus lazertinib as a first-line (MARIPOSA, NCT04487080) and subsequent-line (CHRYSALIS-2, NCT04077463) regimen or in combination with platinum-doublet chemotherapy after progression on osimertinib (MARIPOSA-2, NCT04988295) are ongoing. Another trial is evaluating amivantamab and capmatinib combination therapy in NSCLC patients with *MET* exon 14 skipping mutation and *MET* amplification (METalmark, NCT05488314) (www.clinicaltrial.gov, accessed on 14 May 2023).

Another bispecific antibody binding to two distinct epitopes of MET is REGN5093. This agent is being investigated in a phase 1/2 trial of NSCLC patients with *MET* alterations (with *MET* exon 14 mutations, *MET* gene amplification and MET overexpression) (NCT04077099). Early results showed an acceptable safety profile and promising effectiveness. Grade ≥3 TRAEs occurred in 25% of patients (including pneumonia and pulmonary embolism). In total, 6 patients among the 36 who received the 2000 mg dose had a partial response (2 patients had *MET* exon 14 skipping mutations and 4 patients had *MET* gene amplification and/or overexpression) [93].

Another MET antibody is Sym015, an agent composed of 2 humanized antibodies. These antibodies bind non-overlapping epitopes on the SEMA-a domain of MET leading to MET receptor internalization and degradation avoiding the binding of HGF to MET [94]. Early results from a phase II trial of Sym015 in patients with NSCLC with *MET* amplification or exon 14 skipping mutation (NCT02648724) showed an ORR of 25% and DCR of 80%. For MET TKI-naïve patients, ORR was of 50% and DCR 100% while the median PFS was 6.5 months for the MET TKI-naïve group and 5.4 months for patients who received prior MET TKI therapy. All grade TRAEs occurred in 42.2% of patients, 13.3% of which were grade ≥3 (fatigue and peripheral edema) [94].

#### 4.2.2. Anti-MET Antibody–Drug Conjugates

More recently, antibody–drug conjugates (ADCs) have entered the therapeutic NSCLC scenario with promising early results [95]. Antibody–drug conjugates are constituted by a cytotoxic drug covalently linked to an antibody that targets a specific antigen on tumor cells. Antibody-binding leads to internalization of the ADC-complex with release of the cytotoxic agent and cell death. This unique mechanism allows the release of high doses of chemotherapy to tumor cells, maximizing their antitumor activity while potentially reducing toxicity thanks to the targeted effect [96,97].

Telisotuzumab vedotin is a first-in-class ADC composed of a humanized MET-targeted antibody conjugated to the microtubule inhibitor monomethyl auristatin E (MMAE). On January 2022 the FDA assigned breakthrough therapy designation to telisotuzumab vedotin for advanced, previously treated, *EGFR*-wild-type, nonsquamous NSCLC with MET overexpression, based on the preliminary results of an ongoing phase 2 study evaluating this agent in previously treated patients with MET overexpressing NSCLC (NCT03539536) [98]. Recent interim analysis showed an ORR of 36.5% in patients with *EGFR* wild-type non-squamous subtypes (52.2% and 24.1% in the high and intermediate MET expression groups, respectively). This agent also showed an acceptable safety profile, with the most common TRAEs being dermatitis acneiform, peripheral neuropathy dyspnea, fatigue and hypoalbuminemia [98].

Another ADC composed of a MET antibody conjugated to a novel microtubule inhibitor maytansinoid is REGN5093-M114. This compound is being evaluated in a phase 1/2 trial in patients with MET overexpressing advanced NSCLC and preclinical data are promising (NCT04982224) [99]. An overview of the ongoing clinical trials with anti-MET antibodies in NSCLC is shown in Table 3**.**

## 5. Immunotherapy for Patients with MET Alterations

Limited and conflicting data for use of immunotherapy, in particular of anti-PD1/PD-L1 agents, have been reported in patients with *MET*-altered NSCLC.

This specific molecularly defined subgroup of tumors has been associated with a higher percentage of PD-L1 expression [30,100,101,102]. In a large series of cancers, PD-L1-positive tumors were more frequent in *MET*-altered NSCLC patients (84%) compared with the wild-type group (59%) and especially high PD-L1 expression (50%) was found in the *MET*-altered NSCLC group compared to the wild-type group (60% versus 30%) [30].

A multicenter, retrospective analysis demonstrated an ORR of 16% and a median PFS and OS of 3.4 and 18.4 months, respectively, for ICI therapy in 36 patients with *MET* alterations. This analysis also includes patients with other driver alterations such as *KRAS* (*n* = 271), *EGFR* (*n* = 125), *BRAF* (*n* = 43), *HER2* (*n* = 29), ALK (*n* = 23), *RET* (*n* = 16), *ROS-1* (*n* = 7). Compared to the group with *MET* alterations, ORR was found to be greater in the KRAS (26%), BRAF (24%), and ROS1 (17%) groups, while minor ORR was found in the other groups (EGFR, 12%; HER2, 7%; RET, 6%; and ALK, 0%), while concerning PFS it was found to be lower in the other groups compared to the MET alterations group (2.1 months for EGFR, 3.2 months for KRAS, 2.5 months for ALK, 3.1 months for BRAF, 2.5 months for HER2, 2.1 months for RET) [103]. In another study evaluating a larger number of *MET*-altered NSCLC treated with ICIs, the ORR was 17% and the median PFS 1.9 months [104]. In this study, as in other reports, the tumor mutational burden (TMB) was significantly lower for *MET*ex14-altered versus wild-type tumors (3.8 mutations per megabase versus 7.0 mutations per megabase) [8,105]. Conversely, other retrospective studies have reported an ORR of 36% to 46%, a median PFS of 4.9 months, and durable responses of 10 to 49 months [105,106].

One reason underlying limited immunotherapy efficacy in *MET*-altered NSCLC could be the influence of MET on the tumor microenvironment (TME). MET can modulate TME by making it more immunosuppressive, for example, through hepatocyte growth factor/c-MET signaling resulting in the accumulation neutrophils or by the inhibition of dendritic cells. Therefore, MET inhibition should control this immunosuppression [33,107]. Another reason is through the inhibition of the stimulator of interferon gene (STING) signaling. The STING pathway forwards the interferon (IFN) response and has a significant role in the recruitment of T-cells and NK-cells [108]. A retrospective cohort study, analyzing *MET* copy number and STING levels in patients with NSCLC treated with anti-PD1 therapy following progression on first-line chemotherapy, showed the worst response to treatment in patients with high *MET* copy numbers and low IFN. This suggested that MET amplification leads to reduced tumor immunogenicity thereby minimizing the response to ICI therapy [108].

This suggests a combination of a MET inhibitor with an ICI could potentially overcome resistance to immunotherapy. This strategy is being evaluated in several ongoing trials (NCT04323436, NCT02323126 NCT04797702; NCT03170960 and NCT04471428; NCT03647488; NCT04139317; NCT03468985).

Nevertheless, more data are needed to better assess the role of ICI therapy for treatment of patients with MET alterations, both alone and in combination with targeted drugs.

## 6. Mechanism of Resistance to MET Inhibitors

Despite the initial enthusiasm for the results obtained with the use of MET TKIs in NSCLC patients with MET pathway alterations, both primary and secondary resistance mechanisms, limiting long-term benefit with these agents, have been described [109]. From a molecular point of view, the mechanisms of resistance are similar to those described for other oncogene-directed therapies and can overall be divided into: on-target resistance, which occurs when a genomic alteration prevents the inhibitory activity of the drug on the target protein; off-target resistance, when despite the inhibition of the molecular target, other driver mutations determine tumor growth through alternative pathways; and finally, the third mechanism which involves a histological transformation of the tumor [110].

Several cases of on-target mechanisms have been identified in patients with *MET* alterations treated with MET TKIs and where resistance has been encountered [111]. In most of the patients harboring *MET* exon 14 skipping or *MET* amplification as their primary alteration, secondary mutations in the tyrosine kinase domain of the receptor were identified [6], including mutations in codons G1090, H1094, G1163, L1195, F1200, D1228, Y1230, D1246N, and Y1248H [112].

The type of resistance mutation has been correlated to the type of MET inhibitor used. For example, in patients treated with type Ib inhibitors such as capmatinib, tepotinib, and savolitinib, the most frequent mutations involve codons D1228 and Y1230 [113], while for type II MET inhibitors such as merestinib, glesatinib, and cabozantinib, the resistance mechanism can be due to mutations in codons L1195 and F1200 [111]. Based on this evidence, a resistance mechanism associated with a specific MET TKI could potentially be overcome with the use of a MET TKI belonging to another class [33]. In particular, type Ia inhibitors interact with Y1230 in the MET activation loop, the hinge region and the solvent front residue G1163, while type Ib inhibitors have no interaction with residue G1163. Based on this rationale, the G1163 residue mutation is found almost exclusively in patients treated with MET inhibitors type Ia and where resistance has been encountered but who can however benefit from treatment with type Ib inhibitors. Similarly, type II inhibitors bind to the inactive state of kinases (DFG-out), exploiting interactions inside the lipophilic pocket, and their binding is independent of interactions with the A-loop. In this structure the type II inhibitors have a direct interaction with the F1200 residue that is commonly mutated in patients who have encountered resistance. In this setting, MET inhibitors of type I could overcome the resistance, binding the active state of the kinase [113]. Data on the sequential use of targeted therapies with MET TKIs are still limited but this could represent a valid therapeutic option for patients who develop resistance. In a phase II trial, sequential capmatinib reported modest activity in *MET*-altered NSCLC patients pretreated with crizotinib, potentially due to overlapping resistance mechanisms [114].

Another strategy potentially able to bypass the development of this type of resistance is the use of drugs targeting the extracellular domain of MET. In this regard, a phase 1 study evaluated the efficacy of the monoclonal antibody Amivantamab in patients pre-treated with MET-TKIs, demonstrating efficacy rates in terms of RR 21.1% and clinical benefit in 57.9% [92].

The off-target resistance mechanism is caused by the activation of signal transduction pathways alternative to that of MET. Genomic alterations most frequently associated with this resistance are the amplification of *EGFR* and *KRAS* and the cascade activation of downstream pathways. Preclinical studies, carried out on cell lines obtained from patients treated with MET-TKIs showing resistance due to *KRAS* mutations, evaluated a combination strategy with a MEK1/2 inhibitor (trametinib) and a MET inhibitor (crizotinib). Treatment with trametinib alone showed a low efficacy while the combination was more effective in controlling tumor growth. These results showed that mutation or amplification of *KRAS* can confer resistance to crizotinib treatment in pre-treated *MET* alterations cells and that the addition of trametinib can restore tumor inhibition [115].

Finally, some studies have shown that the activation of the PI3K pathway can also lead to a resistance to MET-TKIs in patients with *MET*-exon14 skipping. These results were consolidated by preclinical studies on patient-derived cell lines treated with a combination of MET and PI3K inhibitors. This combination can inhibit the proliferation of cells harboring a *MET*ex14 mutation and a concurrent PIK3CA mutation or PTEN loss. As *PIK3CA* mutations and *PTEN* loss lead to activation of the PI3K pathway, these results suggest that PI3K pathway alterations cause resistance to MET TKIs [116].

No data are currently available on histological transformation as a mechanism of resistance to MET inhibitors.

### Acquired MET Alterations as a Mechanism of Resistance in EGFR NSCLC

*MET* amplification has emerged as a known mechanism of resistance in a subset of patients (approximately 7–15%) with non-small cell lung cancer (NSCLC) harboring *EGFR* mutations and receiving treatment with EGFR tyrosine kinase inhibitors (TKIs) when experiencing disease progression [117]. These results are in agreement with a seminal preclinical trial by Engelman et al [118], showing that in NSCLC cell lines with *EGFR* mutations, prolonged exposure to EGFR TKIs led to the emergence of *MET* amplification as a resistance mechanism. The cells with *MET* amplification showed sustained activation of the MET signaling pathway, leading to continued cell proliferation and survival even in the presence of EGFR inhibition. Similarly, another preclinical study by Turke et al [119] demonstrated that MET amplification can confer resistance to EGFR TKIs in NSCLC. The researchers established *EGFR*-mutant NSCLC cell lines with acquired resistance to EGFR TKIs and they found that *MET* amplification was present in these resistant cell lines. They also showed that the combination of EGFR and MET inhibitors effectively suppressed cell growth in these resistant cells, highlighting the importance of targeting both pathways to overcome resistance.

These preclinical studies provide evidence that *MET* amplification can arise as a known mechanism of resistance in *EGFR*-mutated NSCLC treated with EGFR TKIs, supporting the need for combination therapies targeting both EGFR and MET pathways to improve treatment outcomes.

In the light of these findings, several studies have investigated the potential of combining MET and EGFR inhibitors. In a phase 1b study, the combination of gefitinib, an anti-EGFR agent, and savolitinib, a highly selective MET inhibitor, demonstrated encouraging objective response rates in patients with different *EGFR* T790M mutation status: 52% (12/23) in T790M-negative patients, 9% (2/23) in T790M-positive patients, and 40% (2/5) in patients with unknown T790M mutation status. Importantly, this combination exhibited a favorable safety profile [120].

In the currently ongoing SAVANNAH phase 2 trial, the efficacy of the combination of osimertinib plus savolitinib is being evaluated. Preliminary data have shown an ORR of 32%, a median DOR of 8.3 months and a median PFS of 5.3 months [121].

The phase II trial INSIGHT investigated antitumor activity of the association of tepotinib plus gefitinib versus standard platinum-doublet chemotherapy in patients with *EGFR*-mutant, T790M-negative NSCLC with MET overexpression or *MET* amplification. Survival outcomes were similar between groups: the median PFS was 4.9 months in the tepotinib plus gefitinib group versus 4.4 months in the chemotherapy group. The median OS was 17.3 months in the tepotinib plus gefitinib group versus 18.7 months in the chemotherapy group. In patients with high (IHC3+) MET overexpression or *MET* amplification, PFS and OS were longer with tepotinib plus gefitinib than with chemotherapy. Respectively, the median PFS was 8.3 months versus 4.4 months and the median OS 37.3 months versus 17.9 months in the MET overexpression group, and the median PFS was 16.6 months versus 4.2 months and the median OS 37.3 months versus 13.1 in the MET amplification group [122]. In the INSIGHT 2 trial, an open-label 2-arm phase 2 study, patients with advanced *EGFR* mutated NSCLC with *MET*-amp after progression on first-line osimertinib received tepotinib 500 mg (450 mg active moiety) + osimertinib 80 mg once daily. First results showed an ORR of 54.5% and the median DOR was not reached among patients with at least 9 months of follow up [123].

In a phase 1b, an antibody–drug conjugate targeting c-MET, Telisotuzumab vedotin (Teliso-V), was also evaluated in combination with erlotinib. The results were promising, with a median PFS of 5.9 months and ORR of 32.1% for EGFR-M+ patients (*n* = 28). Of the EGFR-M+ patients, those who were c-Met high (*n* = 15) had an ORR of 52.6% [124].

Two phase III studies are currently ongoing, evaluating the efficacy of the combination of anti-EGFR and anti-MET. In particular, the SAFFRON study is evaluating the efficacy and safety of savolitinib in combination with osimertinib versus platinum-based doublet chemotherapy in patients with *EGFR* mutated, MET-overexpressed and/or amplified, locally advanced or metastatic NSCLC who have progressed on treatment with osimertinib [86]. Finally, the GEOMETRY-E trial is evaluating the anticancer activity of capmatinib in combination with osimertinib compared to platinum-pemetrexed-based doublet chemotherapy as second-line treatment in patients with NSCLC *EGFR*m, T790M negative, *MET* amplified who progressed following EGFR TKIs (NCT04816214). 

## 7. Conclusions

The role of the MET pathway in NSCLC has been extensively studied, highlighting its significance as both a targetable factor and a mechanism of resistance to other therapies. With advancements in targeted agents, the identification of *MET* exon 14 skipping has become crucial in optimizing the therapeutic approach for advanced NSCLC. However, further data are still needed to determine the most effective sequential strategy among the available MET-TKIs, as well as therapeutic approaches to overcome or delay resistance. Standardizing the detection methods for *MET* alterations is also imperative to ensure consistency and accuracy in clinical practice.

Additionally, several clinical challenges need to be addressed, including the use of MET inhibitors for treating brain metastases and investigating the role of ICIs, either alone or in combination with anti-MET agents, in patients with *MET*-altered NSCLC. These areas require further investigation to develop effective treatment strategies and improve patient outcomes.

## Figures and Tables

**Table 1:** Published trials with MET TKIs in NSCLC.

Drug	Trial	Phase	Treatment	Population	N. of Patients	Results
Crizotinib	PROFILE-1001 (NCT00585195)	I	Crizotinib 250 mg BID	Advanced NSCLC with *MET* exon 14 skipping or *MET* amplification: Low (≥1.8–≤2.2 copies) Medium (≥2.2–≤4 copies) High (> 4 copies)	65 3 14 21	ORR 32% PFS 7.3 months DOR 9.1 months
	METROS (NCT02499614)	II	Crizotinib 250 mg BID	Advanced NSCLC with *MET* exon 14 skipping or *MET* amplification (MET/CEP7 ratio >2.2)	26	ORR 27% PFS 4.4 months DOR 3.7 months
	AcSé (NCT02034981)	II	Crizotinib 250 mg BID	Advanced NSCLC with c-*MET* ≥ 6 copies or all c-*MET*-mutations	25 28	ORR 16%; PFS 3.2 months ORR 10.7%; PFS 2.2 months
Cabozantinib	NCT01866410	II	Cabozantinib 40 mg daily + erlotinib 150 mg daily	Advanced NSCLC with EGFR mutation and progressive disease on EGFR TKI (no *MET* mutations)	37	ORR: 10.8%; PFS: 3.6 months; OS: 13.1 months
	NCT01708954	II	Arm A: erlotinib 150 mg daily Arm B: cabozantinib 60 mg daily Arm C: erlotinib 150 mg + cabozantinib 40 mg	Previously treated advanced NSCLC (*MET* mutations not evaluated)	38 38 35	ORR: 3%; PFS: 1.8 months; OS: 5.1 months ORR: 11%; PFS: 4.3 months; OS: 9.2 months ORR: 3%; PFS: 4.7 months; OS: 13.3 months
Capmatinib	GEOMETRY mono-1 (NCT02414139)	II	Capmatinib 400 mg BID	NSCLC with: *MET* exon 14 skipping (1st line)	28	ORR: 68%; PFS: 12.4 months; DOR: 12.6 months
				*MET* exon 14 skipping (subsequent lines)	69	ORR: 41%; PFS: 5.4 months; DOR: 9.7 months
				*MET* amplification GCN ≥ 10 (1st line)	69	ORR: 29%; PFS: 4.1 months; DOR 8.3 months
				*MET* amplification GCN ≥ 10 (subsequent lines)	15	ORR: 40%; PFS: 4.2 months; DOR 7.5 months
Tepotinib	VISION (NCT02864992)	II	Tepotinib 500 mg once daily	NSCLC with *MET* exon 14 skipping (cohort A)	99	ORR: 46%; PFS: 8.5 months; DOR: 11.1 months;
Savolitinib	NCT02897479	II	Savolitinib 600 mg for BW ≥ 50 kg or 400 mg for BW < 50 kg	Advanced NSCLC or pulmonary sarcomatoid carcinoma with *MET* exon 14 skipping	61	ORR 49.2%; PSF 6.9 months; DCR 93.4%;

Abbreviations: ORR, objective response rate; OS, overall survival; PFS, progression-free survival; DOR, duration of response; DCR, disease control rate.

**Table 2:** Ongoing trials with MET TKIs in NSCLC.

Drug	Trial	Phase	Treatment	Drug Combined	Primary Endpoint (s)	Secondary Endpoint (s)
Crizotinib	Matrix (NCT02664935)	II	Crizotinib 250 mg BD continuous dosing, 21-day cycle	-	OR, PFS, DCB	TTP, OS, Safety
	MATCH (NCT02465060)	II	Crizotinib 250 mg BD on days 1–28	-	ORR	OS, PFS
	NCT04084717	II	Crizotinib 250 mg BD every day of each 28-day cycle	-	ORR, PFS, OS	-
Cabozantinib	NCT01639508	II	Cabozantinib 60 mg every day of each 28-day cycle	-	ORR	PFS, OS, Safety
	LUNG-IST-127 (NCT05613413)	II	Cabozantinib 40 mg once daily Days 1–21 + pembrolizumab 200 mg iv Q3W as maintenance therapy following 4 cycles of induction therapy with disease control	Pembrolizumab	PFS	OS, ORR, Safety
	NCT04310007	II	Arm A: cabozantinib S-malate QD 21-day cycle Arm B: cabozantinib S-malate QD 21-day cycle and nivolumab Q3W Arm C: ramucirumab IV and docetaxel IV Q3W	Nivolumab	PFS	OS, BOR, safety
	CABinMET (NCT03911193)	II	Cabozantinib 60 mg daily (each 28 days)	-	ORR	PFS, OS, DCR, exploratory biomarkers
Merestinib	NCT02920996	II	Merestinib 120 mg daily (each 28 days)	-	ORR	PFS, OS, DOR, Safety
Glesatinib	NCT02544633	II	Glesatinib 750 mg BD	-	ORR	PFS, OS, DOR
	NCT02954991	II	Glesatinib 750 mg BD + Nivolumab 240 mg IV every 2 weeks or 480 mg IV every 4 weeks	Nivolumab	ORR	OS, PFS, Safety
Capmatinb	NCT03693339	II	Capmatinib 400 mg BD continuously dosing	-	ORR	PFS, OS, DOR
	NCT04677595	II	Capmatinib 400 mg BD continuously dosing	-	ORR	DOR, TTR, PFS, OS, IDCR
	NCT05567055	II	Capmatinib 400 mg BD continuously dosing	-	CNS Overall response rate	DOR, PFS, OS, CNS DOR, CNS PFS
	Geometry-N (NCT04926831)	II	Capmatinib 400 mg BD (neoadjuvant/adjuvant setting)	-	MPR	PCR, ORR, DFS, Safety
	NCT04427072	II	Capmatinib 400 mg BD	-	PFS	ORR, DOR, DCR, IDCR, OS
Tepotinib	NCT04739358	I/II	Tepotinib daily in cycles of 21-day duration	Other TKIs	Intracranial ORR, Overall and extracranial ORR	Overall, intracranial and extracranial ORR, PFS, DOR and DCR, safety
	NCT03940703	II	Tepotinib 500 mg once daily and osimertinib 80 mg once daily each 21 day	Osimertinib	ORR, Safety	PFS, OS
	POTENT NCT05782361	I	Tepotinib 500 mg or 250 mg once daily and pembrolizumab 200 mg Q3W	Pembrolizumab	Antitumor activity	Safety, tolerability
Savolitinib	NCT04923945	III	Savolitinib 600 mg once daily continuously in patients with BW ≥ 50 kg and Savolitinib 400 mg once daily in patients with BW < 50 kg	-	ORR	PFS, Safety
	NCT05777278	I/II	Savolitinib 300 mg or 200 mg BD and Docetaxel 60 mg/m^2^ iv Q3W	Docetaxel	ORR	PFS, OS, DCR, DOR
	NCT05374603	II	Savolitinib 600 mg for BW ≥ 50 kg, 400 mg for BW < 50 kg once daily and Durvalumab 1500 mg iv Q4W	Durvalumab	PFS	ORR, DOR, DCR, OS
	SAVANNAH NCT03778229	II	Savolitinib 300 mg once daily or 300 mg BD or 600 mg once daily and osimertinib 80 mg oral once daily	Osimertinib	ORR	PFS, OS, DOR
	SAFFRON NCT05261399	III	Arm A: Pemetrexed (500 mg/m^2^) with either cisplatin (75 mg/m^2^) or carboplatin (AUC5) Q3W for 4 cycles, followed by pemetrexed maintenance (500 mg/m^2^) Q3W Arm B: Savolitinib 300 mg BD + osimertinib 80 mg once daily	Osimertinib	PFS	OS, ORR, DCR, DOR
	ORCHARD NCT03944772	II	Savolitinib 300 mg or 600 mg once daily + Osimertinib 80 mg once daily	Osimertinib	ORR	PFS, DOR, OS
	SACHI NCT05015608	III	Savolitinib once daily + Osimertinib once daily (every 3 weeks)	Osimertinib	PFS	ORR, OS, DOR, DCR, TTR, Safety
	FLOWERS NCT05163249	II	Savolitinib 300 mg BD+, Osimertinib 80 mg once daily	Osimertinib	ORR	PFS, DOR, DCR, OS
	SANOVO NCT05009836	III	Savolitinib 600 mg or 400 mg once daily + Osimertinib 80 once daily (every 3 weeks)	Osimertinib	PFS	ORR, OS, DOR, DCR, TTR, Safety
APL-101	SPARTA NCT03175224	I/II	APL-101 28-day cycles at four planned dose levels (100 mg, 200 mg, 300 mg and 400 mg)	-	Safety, ORR	ORR, DOR, PFS, TTP
Elzovantinib or TPX-0022	SHIELD-1 NCT03993873	I/II	Elzovantinib	-	Safety, tolerability	PFS, OS, ORR, DOR, TTR
Glu*MET*inib or SCC244	GLORY, NCT04270591	I/II	Glumetinib 300 mg once daily	-	ORR	DOR, OS

Abbreviations: ORR, objective response rate; OS, overall survival; PFS, progression-free survival; DOR, duration of response; DCR, disease control rate; DCB, durable clinical benefit; TTP, time to progression; CNS, central nervous system; BOR, best objective response; TTR, time to response; IDCR, intracranial disease control rate.

**Table 3:** Ongoing trials with anti-MET antibodies in NSCLC.

Drug	Trial	Phase	Treatment	Drug Combined	Primary Endpoint (s)	Secondary Endpoint (s)
Amivantamab	CHRYSALIS NCT02609776	I	Amivantamab + Lazertinib	Lazertinib	Safety, DLT, ORR, DOR	PFS, TTF, OS
	CHRYSALIS-2 NCT04077463	I/Ib	Amivantamab + Lazertinib	Lazertinib	DLT, ORR, Safety, DOR	PFS, TTF, OS, Intracranial PFS
	MARIPOSA NCT04487080	III	Arm A: Amivantamab 1050 mg iv BW less than <80 kg and 1400 mg for BW ≥ 80 kg in 28-day cycles + Lazertinib 240 mg once daily Arm B: osimertinib 80 mg o once daily + lazertinib 240 mg once daily and placebo Arm C: lazertinib 240 mg once daily+ osimertinib 80 mg once daily and placebo	Lazertinib	PFS	ORR, OS, DOR, TTP, PFS2, intracranial PFS, Safety
	MARIPOSA-2 NCT04988295	II	Arm A: Amivantamab + Lazertinib, Pemetrexed and Carboplatin (LACP dosing or ACP-L dosing) Arm B: Pemetrexed + Carboplatin iv for up to 4 cycles Q3W → Pemetrexed as maintenance until progression Arm C: Amivantamab+ Pemetrexed + Carboplatin iv for up to 4 cycles Q3W → Amivantamab + Pemetrexed as maintenance until progression	Lazertinib, pemetrexed, carboplatin	PFS	ORR, OS, DOR, TTST, PFS2
	METalmark NCT05488314	I/II	Amivantamab 700 mg iv for BW less than 80 kg or 1050 mg for BW greater than or equal to 80 kg + Capmatinib 400 mg twice daily	Capmatinib	DLT, Safety, ORR	PFS, DOR, OS, Safety
REGN5093	NCT04077099	I/II	REGN5093 iv Monotherapy in dose escalation cohorts followed by an expansion phase	-	*DLT*, safety, ORR	PFS, OS, DOR, DCR
Sym015	NCT02648724	I/II	Sym015 at different dose levels 6, 12, 18, and 24 mg/kg.	-	DLT, ORR	AUC, Tmax
Telisotuzumab vedotin	NCT03539536	II	Telisotuzumab vedotin iv infusion every 14 days	-	ORR, safety	OS, PFS, DOR, DCR
REGN5093-M114	NCT04982224	I/II	REGN5093-M114 Iv infusion	-	DLT, Safety	ORR, DCR, PFS, OS, DOR, TTR

Abbreviations: ORR, objective response rate; OS, overall survival; PFS, progression-free survival; PFS2 progression-free survival 2; DOR, duration of response; DCR, disease control rate; DLT, dose limiting toxicity; BOR, best objective response; TTP, time to progression; TTF, time to treatment failure; TTST, time to subsequent therapy; TTR, time to tumor response; AUC, area under the concentration-time curve; Tmax, time to reach maximum concentration.

## Data Availability

Not applicable.
